# An Ultra-wideband and Polarization-independent Metasurface for RCS Reduction

**DOI:** 10.1038/srep20387

**Published:** 2016-02-11

**Authors:** Pei Su, Yongjiu Zhao, Shengli Jia, Wenwen Shi, Hongli Wang

**Affiliations:** 1 Key Laboratory of Radar Imaging and Microwave Photonics, Ministry of Education, Nanjing University of Aeronautics and Astronautics, Nanjing 210016, China

## Abstract

In this paper, an ultra-wideband and polarization-independent metasurface for radar cross section (RCS) reduction is proposed. The unit cell of the metasurface operates in a linear cross-polarization scheme in a broad band. The phase and amplitude of cross-polarized reflection can be separately controlled by its geometry and rotation angle. Based on the diffuse reflection theory, a 3-bit coding metasurface is designed to reduce the RCS in an ultra-wide band. The wideband property of the metasurface benefits from the wideband cross polarization conversion and flexible phase modulation. In addition, the polarization-independent feature of the metasurface is achieved by tailoring the rotation angle of each element. Both the simulated and measured results demonstrate that the proposed metasurface can reduce the RCS significantly in an ultra-wide frequency band for both normal and oblique incidences, which makes it promising in the applications such as electromagnetic cloaking.

Metamaterials are composed of periodic or non-periodic unit cells which are much smaller than a wavelength^1^,^2^,^3^. Due to their unusual electromagnetic properties, metamaterials can be utilized for manipulating light propagation, such as negative refraction^4^,^5^,^6^,^7^,^8^,^9^, spatial localization and sub-wavelength focusing^5^, spontaneous emission control^9^,^10^, and anomalous tunneling effects^11^,^12^,^13^. One important application of metamaterial is electromagnetic cloaking that can reduce the RCS of metallic or dielectric targets. The method of optical transformation provides an efficient way to bend the electromagnetic wave around a given region and make it effectively invisible^14^,^15^,^16^. Another viable technique is plasmonic cloaking based on scattering cancellation, where a homogeneous layer of metamaterial is designed to produce a local polarization vector in anti-phase with respect to that of the cloaked object^17^,^18^,^19^,^20^. Both of the above cloaking techniques rely on the electromagnetic properties of bulk metamaterials. Considerable thickness and weight of the material are required to achieve a desirable cloaking effect, which also increases fabrication challenges. This has led to the development of the two-dimensional equivalence of metamaterials called metasurfaces. Mantle cloak is a promising method that utilizes metasurfaces to achieve invisibility by wrapping the objects and inducing suitable surface currents to generate anti-phase scattered fields^21^,^22^,^23^,^24^. But this technique works in a narrow frequency band and is only suitable for regular shaped structures smaller than a wavelength. The radar absorbing metamaterial/metasurface can also be used for RCS reduction by transforming electromagnetic energy into heat^25^,^26^,^27^. However, radar absorbing metamaterials usually operate in the vicinity of resonance frequency. Chessboard-like structure composed of perfect electronic conductor (PEC) and artificial magnetic conductor (AMC) has been proposed to reduce mirror reflection^28^,^29^,^30^. However, broadband AMC structures are difficult to design and implement. Therefore, extending the operating bandwidth of the metasurface for RCS reduction remains a challenge.

In this paper, an ultra-wideband and polarization-independent metasurface is proposed for RCS reduction. The unit cell of the proposed metasurface is capable of rotating a linearly polarization to its orthogonal one. Multiple resonances are generated on the unit cell, resulting in the bandwidth expansion of cross polarization conversion. The phase and amplitude of cross-polarized reflection can be separately controlled by the open angle and rotation angle of the unit cell. A 3-bit coding metasurface, consisting of eight types of unit cells with different open angles, is designed based on the diffuse reflection theory. The ultra-wideband RCS reduction feature of the metasurface benefits from both the wideband linear polarization conversion property and flexible phase modulation ability of the unit cell. Furthermore, the polarization-independent feature of the metasurface can be achieved by adjusting the rotation angle of each element. Both simulated and measured results indicate that the proposed metasurface can significantly reduce the RCS from a bare metal plate in an ultra-wide frequency band for both normal and oblique incidences. The co-polarized RCS reduction is more than 10 dB in 7.9–20.8 GHz for normal x- and y-polarized incident waves. Under oblique incidences, the bandwidth decreases slightly due to phase aberrations. Nevertheless, the proposed metasurface still performs well in the operating band.

## Results

### Unit cell design

**Figure 1:**
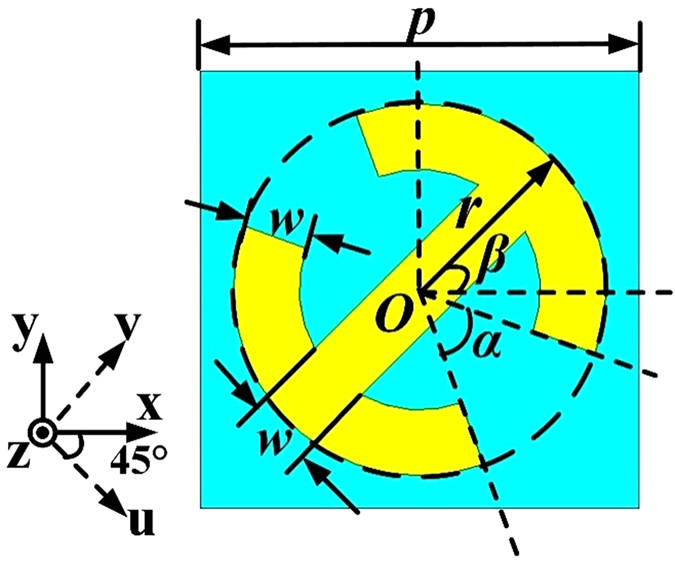
The front view of the unit cell. The point O is the origin of coordinates. *p* = 10 mm, *r* = 4.25 mm, *w* = 1.5 mm. *α* represents the open angle of the symmetric split ring and *β* is the rotation angle of unit cell. U- and v-axes are along 45° direction with respect to x and y direction.

The front structure of the unit cell is depicted in Fig. 1. The structure, composed of a symmetric split ring and a cut wire, is patched on the substrate F4B (thickness *h* = 3.0 mm, dielectric constant 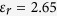, loss tangent 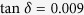). Other dimensions of the structure are listed in Fig. 1, where 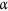 represents the open angle of the symmetric split ring, *β* is the rotation angle of the unit cell. *α* and *β* will be mainly considered in the following discussions. For the sake of analysis, u- and v-axes are introduced here along 45° direction with respect to x and y directions.

**Figure 2:**
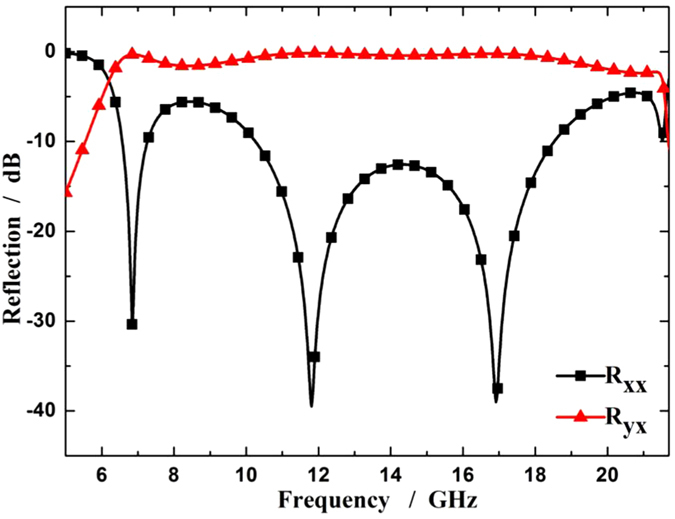
The simulated reflections of the unit cell under normal x-polarized incident waves.

The simulations of the unit cell are accomplished with commercial software CST Microwave Studio, with periodic boundary conditions in x and y directions and floquet ports in z direction. Fig. 2 shows the simulated reflection of the unit cell for normal x-polarized incidence, with *α* to be 90° and *β* to be 45°. In the figure, R_xx_ and R_yx_ represent the reflections of the co- and cross-polarized waves, respectively. It can be seen that cross-polarized reflection is strong over a broad band under the incidence of normal x-polarized waves. Actually, the ultra-wideband property results from multiple resonant modes on the unit cell. The symmetric split ring supports symmetric and anti-symmetric modes excited by electric-field components along v- and u-axes^31^,^32^, whereas the cut wire supports multi-order modes excited by electric-field components along v-axis. As the combination of the two structure, the unit cell can realize multiple resonances at different frequencies, leading to the broadband performance of the linear polarization conversion.

**Figure 3:**
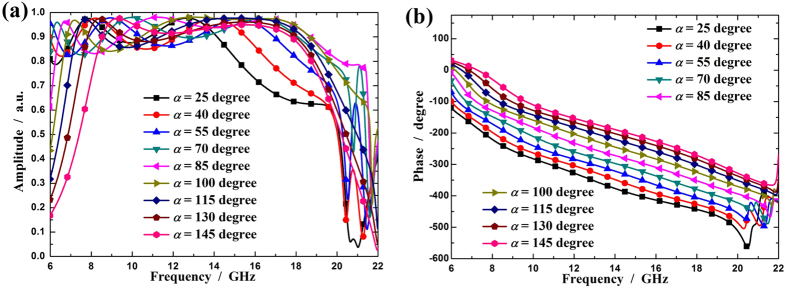
The cross-polarized reflection coefficients for different open angles. (**a**) Amplitude and (**b**) phase of cross-polarized reflection coefficient for different *α* values under normal x-polarized incident waves.

Figure 3 gives the cross-polarized reflection coefficients for different *α* values and fixed 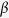 (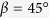). As shown in Fig. 3(a), when the open angle varies from 25° to 145°, the amplitudes of cross-polarized reflections are greater than 0.8 in a broad band. From Fig. 3(b), it can be seen that the linear response of the phase is not affected, except for a constant phase shift due to different *α* values. The phase shift covers a range of more than 180° over a wide frequency when *α* changes from 25° to 145°. Due to symmetry, a mirror structure (with respect to y-axis) of the unit cell can produce the same cross-polarized reflection except for a 180° phase shift. As a result, with both structures, full control of the phase of cross-polarized reflection can be achieved while amplitude remains substantially constant.

**Figure 4:**
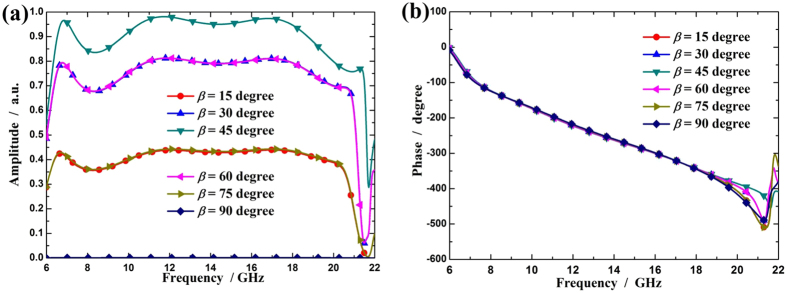
The cross-polarized reflection coefficients for different rotation angles of the unit cell. (**a**) Amplitude and (**b**) phase of cross-polarized reflection coefficient for different *β* values under normal x-polarized incident waves.

Figure 4 shows the cross-polarized reflection coefficients for different *β* values and fixed *α* (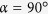). It is clearly that the amplitude of cross-polarized reflection can be continuously controlled in a broad band by adjusting the rotation angle while the phase remains constant.

In general, both the phase and amplitude of cross-polarized reflection can be controlled within a wide band by separately tailoring the open angle and rotation angle of the unit cell. This greatly facilitates the complete control of light propagation and the realization of RCS reduction.

### Metasurface design and simulations

**Figure 5:**
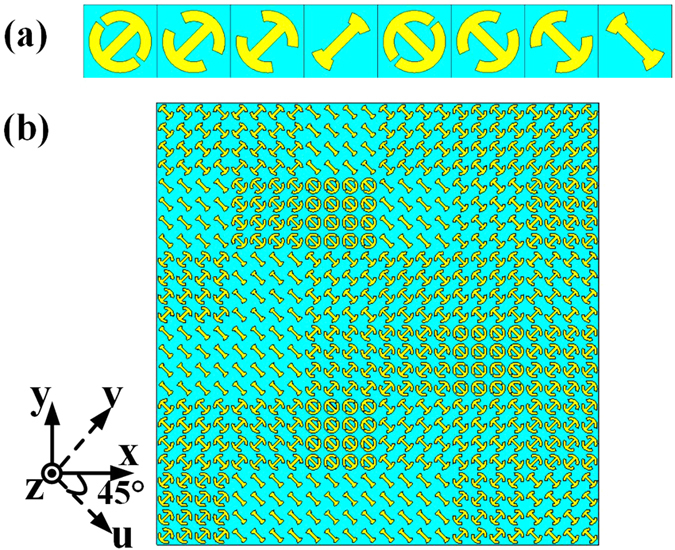
Eight basic unit cells and the 3-bit coding metasurface. (**a**) Eight basic unit cells with the same *p* = 10 mm, *r* = 4.25 mm, *w* = 1.5 mm but different 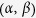 = (10.0°, 45°), (41.4°, 45°), (67.8°, 45°), (132.0°, 45°), (10.0°, −45°), (41.4°, −45°), (67.8°, −45°), (132.0°, −45°), corresponding phase responses of 0, 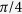, 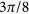, 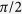, 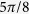, 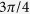 and 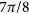 to mimic the ‘000’, ‘001’, ‘010’, ‘011’, ‘100’, ‘101’, ‘110’ and ‘111’ elements, respectively. *β* is selected to be ±45° for maximizing the conversion between x and y polarizations. (b) The 3-bit coding metasurface structure, containing 6 × 6 equal-sized lattices. The period of the each lattice is 40 mm and each lattice is composed of 4 × 4 elements with the same dimension.

**Figure 6:**
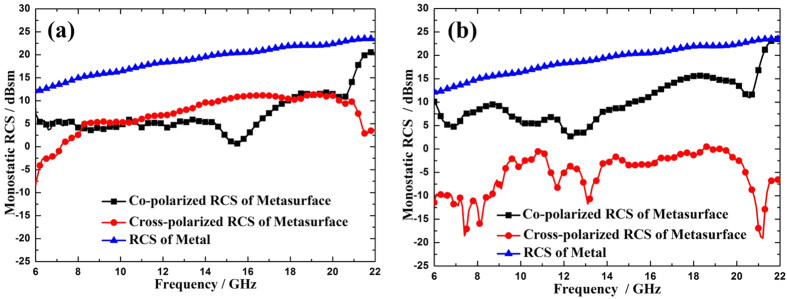
The simulated RCSs for the 3-bit coding metasurface and a metal plate with the same dimension. (**a**) Under normal x-polarized incident waves. (**b**) Under normal v-polarized incident waves.

The operating mechanism of our proposed metasurface is to diffuse the scattered energy into many directions using random distribution of the reflection phases, and as a result, dramatically reduce the backward scattering^33^,^34^. The random distribution of the reflection phases is designed using eight types of unit cells, which conforms to a 3-bit coding scheme extended from ref. 35. The eight types of unit cells have phase responses of 0, 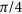, 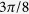, 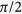, 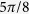, 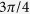, and 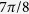, which mimic the ‘000’, ‘001’, ‘010’, ‘011’, ‘100’, ‘101’, ‘110’ and ‘111’ elements, respectively. The dimensions of the eight basic elements, as given in Fig. 5(a), are derived from the relation between the open angle *α* and the cross-polarized reflection phase at 14 GHz. Then by coding the elements with a random sequence, the whole metasurface is constructed, as shown in Fig. 5(b). When x-polarized waves are normally incident on the metasurface, the polarization of a substantial component of the scattered field is orthogonal to that of the incident wave (Fig. 6(a)). The reason is that both the symmetric and anti-symmetric modes are excited since x-polarized waves have both u- and v-components simultaneously, maximizing the conversion between x and y polarization. However, when the metasurface is illuminated by normal v-polarized incident waves, only a small component of incident waves are converted to the cross polarization, as shown in Fig. 6(b). Therefore, the 3-bit coding metasurface shown in Fig. 5(b) is polarization dependent.

**Figure 7:**
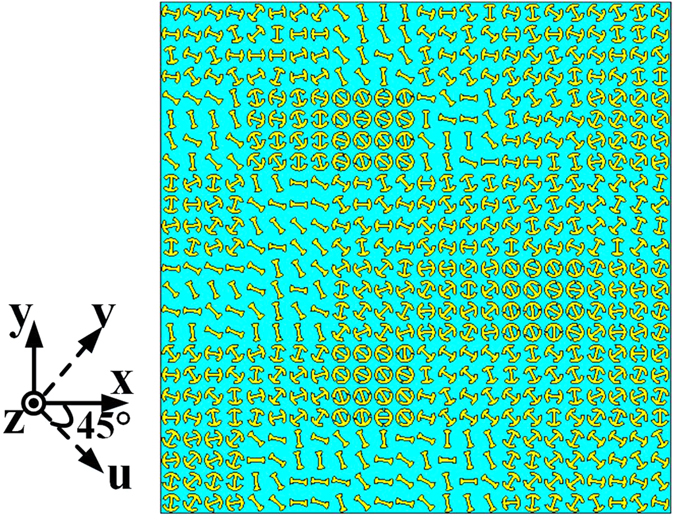
The random 3-bit coding metasurface. The rotation angle of each element is tailored randomly in the range of 0°–360° to construct the random metasurface.

**Figure 8:**
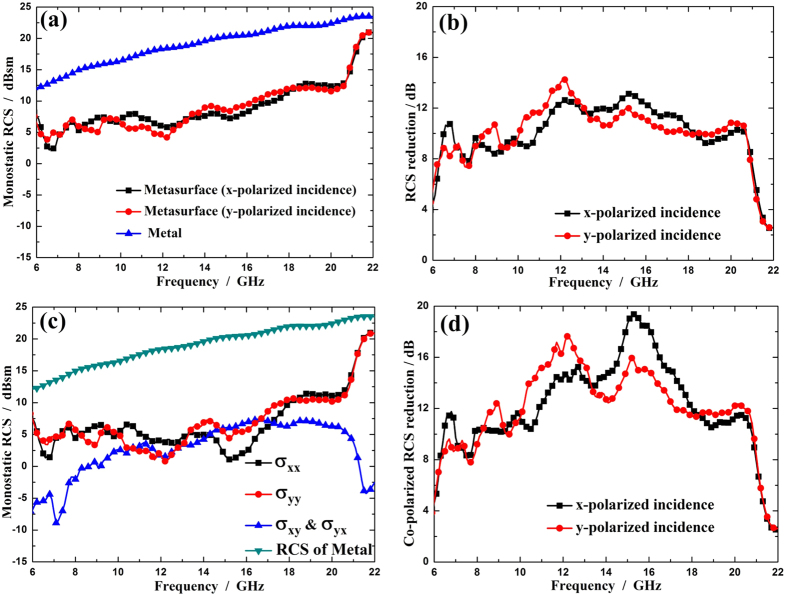
The simulated results of the random 3-bit coding metasurface under normal incident waves. (**a**) The simulated RCS and (**b**) RCS reduction for normal x- and y-polarized incident waves. (**c**) The simulated RCSs for four transmit-receive polarization cases and (**d**) co-polarized RCS reduction for normal x- and y-polarized incident waves. The RCS of a metal with the same dimension as the metasurface is also given in (**a**) and (**c**) for comparison.

In order to realize RCS reduction for arbitrary linear polarized waves, the rotation angle of each element is tailored randomly in the range of 0°–360° to construct a random 3-bit coding metasurface (see Fig. 7). As discussed previously, the phase property of cross-polarized wave is not influenced by the change of the rotation angle. Fig. 8(a) shows the monostatic RCS of a bare metal plate and the metal plate coated with the metasurface for normal x- and y-polarized incident waves. It can be seen that the backward scattering from a metal plate can be reduced significantly by covering it with the designed metasurface. As demonstrated in Fig. 8(b), the RCS is suppressed by 8 dB from 7.9 to 21 GHz for both x- and y-polarized incident waves. Fig. 8(c) shows monostatic RCSs of the metasurface for four transmit-receive polarization cases under normal incidences, where the RCS of the metal plate is also given for comparison. In the figure, 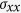, 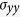 represent co-polarized RCS (with the incident and backscattering waves in the same polarization), and 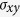, 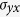 represent cross-polarized RCS (with the incident and backscattering waves in different polarizations). It is found that both co- and cross-polarized RCS of the metasurface are much less than the monostatic RCS of the metal plate. Furthermore, the co-polarized RCS of the metasurface is close to the cross-polarized RCS, indicating that about half of incident energy is converted to cross polarization. It is worth mentioning that the proposed metasurface shows good polarization conversion performance for both x- and y-polarized incident waves, which demonstrates the polarization independence of the metasurface. As depicted in Fig. 8(d), the co-polarized RCS reduction is over 10 dB in 7.9–20.8 GHz, which greatly decreases the detection possibility of the target by linear polarization radar.

### Experimental results

**Figure 9:**
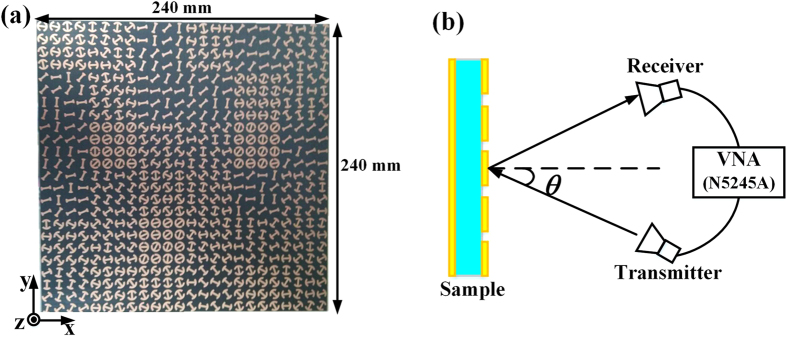
The measurement of the metasurface. (**a**) The fabricated metasurface and (**b**) the schematic of measurement setup.

**Figure 10:**
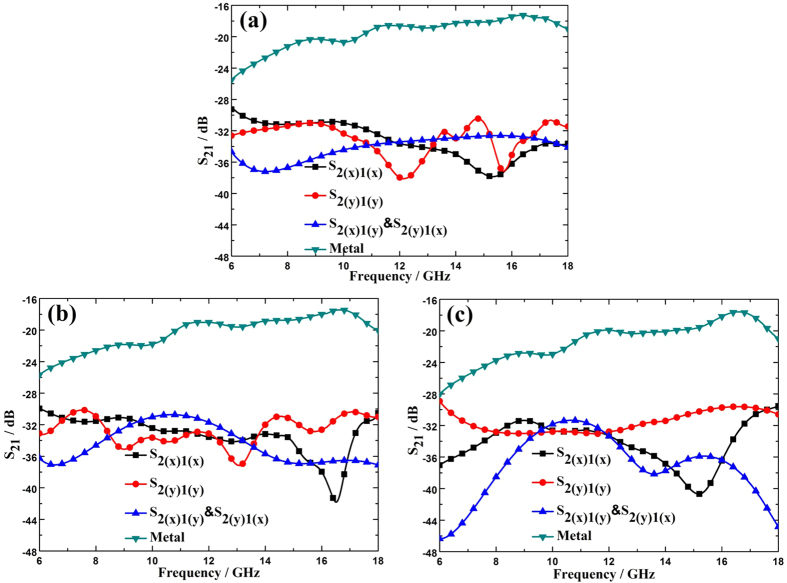
The measured scattering coefficients from a bare metal plate and the metasurface sample with a vector network analyzer (Agilent N5245A). (**a**) The incident angle *θ* = 0 degree, (**b**) *θ* = 15 degree, (**c**) *θ* = 30 degree.

**Figure 11:**
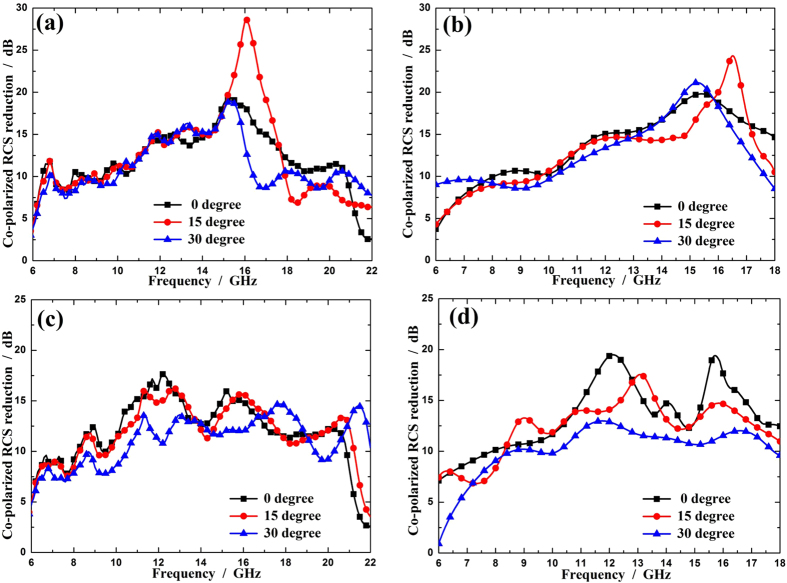
The measured co-polarized RCS reduction. (**a**) Simulated and (**b**) experimental co-polarized RCS reduction for various incident angles with x polarization. (**c**) Simulated and (**d**) experimental co-polarized RCS reduction for various incident angles with y polarization.

To further verify the design, a sample (240 mm × 240 mm) of the metasurface is fabricated, as depicted in Fig. 9(a). To obtain the RCS reduction, both the scattering coefficients from a bare metal plate and the metasurface sample are measured. The experiment setup is illustrated in Fig. 9(b), where two antennas are connected to a vector network analyzer (Agilent N5245A). When rotated by 90°, the horn antennas can be reconfigured between transverse-magnetic (TM) and transverse-electric (TE) modes, so that both the co- and cross- polarized scattering can be measured. In addition, the scattering properties of the metasurface under oblique incidences are evaluated by varying the incident angle 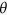 from 0° to 30°. Due to the limitations of experimental conditions, the measurement is conducted only in the range of 6–18 GHz. The measured scattering coefficients from a bare metal plate and the metasurface sample at different incident angles are illustrated in Fig. 10. As expected, the resulting co-polarized scattering of the metasurface has a magnitude comparable to that of the cross-polarized scattering for different incident angles, verifying the polarization conversion property of the metasurface. After some mathematical manipulating, the co-polarized RCS reduction is achieved for both x and y polarizations as shown in Fig. 11, from which it can be seen that the metasurface can effectively decrease the co-polarized RCS from the bare metal plate. For normal incidence, over 10 dB co-polarized RCS reduction is achieved over a broad band from 8 GHz to 18 GHz. As the incident angle grows, the bandwidth decreases a little due to the phase aberrations. However, the proposed metasurface still performs well in the operating band.

## Discussions

In this paper, a metasurface for RCS reduction has been designed, fabricated and measured. The random 3-bit coding metasurface, consisting of eight types of unit cells with different open angles, is designed based on the diffuse reflection theory. The ultra-wideband RCS reduction feature of the metasurface benefits from both the wideband linear polarization conversion property and flexible phase modulation ability of the unit cell. Furthermore, the polarization-independent feature of the metasurface is achieved by adjusting the rotation angle of each element. Both the simulated and measured results demonstrate that the metasurface can effectively reduce the RCS from the bare metal plate in an ultra-wide band. In comparison to previous approaches, our metasurface for RCS reduction has the advantage of easy fabrication, broad bandwidth and polarization independence of incident waves, which makes it promising for electromagnetic cloaking.

## Methods

To obtain the RCS reduction, both the scattering coefficients from a bare metal plate and the metasurface sample are measured. The measurements are carried in a microwave anechoic chamber. The sample is placed as high as the horn antennas in the experiment. The distance between antennas and sample is chosen far enough to avoid the near field effect. Two standard linearly polarized horn antennas serve as transmitter and receiver, respectively. The antennas are connected to a vector network analyzer (Agilent N5245A), which has the function of a time domain gating. When rotated by 90°, the horn antennas can be reconfigured between transverse-magnetic (TM) and transverse-electric (TE) modes, so that both the co- and cross- polarized scattering can be measured. In the case of normal incidence, the transmitting and receiving horn antennas are placed adjacently. Both the transmitting and receiving horn antennas can move along the circumference trace to obtain the scattering at different incident angles.

## Additional Information

**How to cite this article**: Su, P. *et al.* An Ultra-wideband and Polarization-independent Metasurface for RCS Reduction. *Sci. Rep.* **6**, 20387; doi: 10.1038/srep20387 (2016).
